# Predictive Role of the Number of ^18^F-FDG-Positive Lymph Nodes Detected by PET/CT for Pre-Treatment Evaluation of Locally Advanced Gastric Cancer

**DOI:** 10.1371/journal.pone.0166836

**Published:** 2016-12-09

**Authors:** Xin Wang, Yuzhe Wei, Yingwei Xue, Peiou Lu, Lijuan Yu, Baozhong Shen

**Affiliations:** 1 PET/CT-MRI Centre, Harbin Medical University Cancer Hospital, Nangang District, Harbin, Heilongjiang Province, China; 2 Department of Gastroenterological Surgery, Harbin Medical University Cancer Hospital, Nangang District, Harbin, Heilongjiang Province, China; 3 Department of Radiology, Fourth Hospital of Harbin Medical University, Nangang District, Harbin, Heilongjiang Province, China; University of Crete, GREECE

## Abstract

**Objectives:**

The aim of this study was to investigate the predictive value of the numbers of metabolically positive lymph nodes (MPLN) detected by ^18^F-fluorodeoxyglucose (^18^F-FDG) positron emission tomography (PET)/computed tomography (CT) in patients with locally advanced gastric cancer (LAGC).

**Methods:**

We retrospectively analyzed the records of 50 patients with LAGC (stage T2-T4) who had undergone pre-operative PET/CT examination and laparotomy (total gastrectomy, n = 11; subtotal gastrectomy, n = 13; distal gastrectomy, n = 22; and bypass with gastrojejunstomy, n = 4). The numbers of MPLN were determined by combining visual observations with semi-quantitative measurements of the maximized standardized uptake value (SUV_max_). Performance was investigated in terms of predicting post-surgical overall survival (OS).

**Results:**

The median post-surgical OS was 32.57 months (range 3.0-94 months). The numbers of MPLN were moderately correlated with the numbers of histological positive LN (r = 0.694, p = 0.001). In univariate analyses, the numbers of MPLN (≤ 2 *vs*. ≥3), PET/CT LN (positivity *vs*. negativity), SUV_max_ of LN (< 2.8 *vs*. ≥ 2.8), TNM stage (I, II *vs*. III, IV), and surgery type (R0 *vs*. non-R0) were significantly associated with OS. In multivariate analysis, surgery type (R0 vs. non-R0) and numbers of MPLN (≤ 2 vs. ≥ 3) were both independent factors for poor OS.

**Conclusions:**

This explored study indicates that the number of MPLN could provide additional information for LAGC prognosis. Patients with MPLNs ≥ 3 may be at the risk of the more bad outcomes, and the further clinical trials are needed.

## Introduction

Gastric cancer is a common malignancy worldwide, and has the second greatest incidence and mortality rates of malignancies in China [1]. Only about 20% of gastric cancers are diagnosed at an early stage in the Chinese population, and the majority are diagnosed in advanced stages. Surgery is the most important and only therapy that has curative potential for gastric cancer, but benefits varies. For local advanced gastric cancer (LAGC), the postoperative five-year survival rate is still low. In recent years, with development of preoperative chemotherapy, targeted drugs, and immunotherapy, gastric cancer treatment is moving into the diversified era [2–4]. Therefore, information on patients who do or do not achieve long-term survival through surgery would be important for optimizing treatment plans.

^18^F-fluorodeoxyglucose (^18^F-FDG) PET/CT is a functional modality that can present glucose-metabolic activity of the vivid tissue. Currently, metabolic parameters derived from ^18^F-FDG PET/CT, including standardized uptake value (SUV), metabolic tumor volume (MTV), and total lesion glycolysis (TLG), have demonstrated prognostic value in multi-malignancy tumours, such as head and neck cancer, pancreatic cancer, non-small cell lung cancer, etc. [5–7]. There are few reports on these parameters’ predictive values for gastric cancer [8–12]. Two studies found that high SUV_max_ of primary lesions measured on pretreatment ^18^F-FDG PET/CT predict poor clinical outcome for patients with metastatic advanced gastric cancer who undergo palliative chemotherapy [8, 9]. The prognostic value of primary SUV for resectable gastric cancer was not been well established [10–12].

Recently there has been research on the predicted value of ^18^F-FDG PET/CT positive lymph nodes for gastric cancer. Hur et al. [12] reported that high SUV of the primary tumour (>5) and positive ^18^F-FDG uptake in local lymph nodes during PET/CT could predict surgical failure to cure LAGC, but sensitivity and positive predictive values were low, 35.2% and 57.1%, respectively. Coupe et al. [13] and Song et al. [14] reported that lymph node positivity or high SUV indicated by PET was an independent predictor for inferior OS. These findings suggest that metabolically positive lymph nodes (referred to as “MPLN”) detected by PET/CT may be a significant marker for gastric cancer.

In this study, we retrospectively analysed data from a group of patients with LAGC (T2-4) who had undergone surgery but not neoadjuvant therapy to investigate the predictive performance of MPLN detected by preoperative ^18^F-FDG PET/CT. In addition to SUV of lymph nodes, we counted the numbers of MPLN as another parameter to explore whether numbers of MPLN can provide additional information for LAGC post-surgical OS or contribute to classifying patients’ prognosis.

## Materials and Methods

### Patients

The study was approved by the Ethics Committee of the Harbin Medical University Cancer Hospital. We retrospectively reviewed the computerized medical records of 241 patients with gastric cancer who had undergone pre-treatment ^18^F-FDG-PET/CT and surgical therapy between January 2008 and October 2013. We excluded early gastric cancer (T1), squamous cell carcinoma of the gastroesophageal junction, distant metastatic gastric cancer, undergoing neoadjuvant therapy before PET/CT examination or surgery, and those with a history of diabetes or a second primary tumour. Patients with no evidence of distant metastases until discovered during laparotomy were included. This rendered a total of 50 patients with advanced gastric adenocarcinoma (T2-T4) being enrolled in this study. These patients were treated by a single surgery team with extensive experience with radical resection for gastric carcinoma.

### Treatment and follow-up

All patients underwent laparotomy. Abdominal and pelvic cavities and organs were examined according to the disease-free principle of far to near, followed by biopsies and quick-frozen pathology of lesions suspicious for distant metastasis. Surgical approach was then determined. Eleven underwent total gastrectomy, and one with bloc pancreaticoduodenectomy. Of the 50 patients, 35 underwent subtotal or distal gastrectomy, and four underwent palliative bypass surgery for peritoneal metastasis (n = 3) and pancreatic metastasis (n = 1). D2 lymphadenectomy was performed for 12 patients, and D2 lymphadenectomy plus hepatoduodenal ligament lymph node and mesenteric artery lymph node dissection was performed for 34, among whom three had para-aortic lymph node biopsies and two of them were verified as metastatic nodes by pathology.

Surgeons and pathologists confirmed no post-operative residual tumour, namely R0 resection (n = 39), and surgeons confirmed possible tumour residue in the pancreatic capsule (n = 2) and the transverse mesocolon root (n = 3). Post-operative staging was according to the TNM staging method specified in the NCCN Gastric Cancer Guidelines (2013) [15]. Gastric cancer pathology types and grades were according to WHO categorization. Any serum tumour markers (CEA/CA19-9/CA72-4) that had higher than normal levels before surgery were recorded as tumour marker-positive.

All patients received adjuvant chemotherapy (2–6 cycles) at baseline with 5-FU cell toxic drugs post- surgery. OS was calculated from the date of surgery to the date of death or the end point of follow-up. Thirty-four patients had completed regular follow-up records, including laboratory tests and imaging examination data, 13 had incomplete follow-up records with patient survival information obtained by telephone, and three were lost to follow-up.

### ^18^F-FDG PET/CT imaging protocol

All patients underwent imaging with two different integrated scanners, Discovery ST and Discovery^™^ Elite (GE Medical Systems, Inc., Waukesha, WI, USA). Prior to PET/CT examination, patients fasted for at least six hours. After measurement of their fasting blood glucose, height, and weight, the patients received an intravenous injection of ^18^F-FDG of approximately 3.7 MBq/kg. This was followed by bed rest for 60 min, during which the patient was asked to drink 500 ml water or 2% diatrizoate aqueous solution twice at 30-min intervals to distend the gastric cavity and small intestine. Patients were placed in a supine position and asked to keep their breathing steady while undergoing low-dose CT examination (120kV, 80–160mAs) from skull to upper femur. Subsequent PET acquisition was performed with five to seven table positions covering the same range and a scanning time of 2.5 min/bed when using Discovery ST, for which 2D reconstruction techniques were employed, and 1.5 min/bed when using Discovery^™^ Elite, for which 3D reconstruction techniques were employed. CT data were used to perform attenuation correction. The standard uptake value (SUV) was automatically calculated by the device according to the patient’s height, weight, and blood sugar levels, and the injection dose. Patients whose gastric cavity was under-distended or was not consistent with PET images from the first scan underwent additional two-hour delayed imaging after drinking a further 500 ml of 2% diatrizoate aqueous solution.

### Measurement of metabolic parameters of ^18^F-FDG PET/CT

^18^F-FDG-PET/CT images were interpreted by two radiologists with more than five years of PET/CT experience, each of whom was unaware of the case’s postoperative information. In cases of discrepancy, consensus was reached and used for analysis.

For the primary lesion, ^18^F-FDG uptake was assessed through visual inspection of both the coronal and transverse PET images. The lesions that exhibited higher ^18^F-FDG uptake than the liver were recorded as positive cases, and the lesions that showed ^18^F-FDG uptake levels similar to the liver or could not be differentiated from the normal stomach wall were defined as negative cases. The maximum SUV (SUV_max_), when corrected for body weight, was measured from the highest uptake point of the lesion on transverse PET images.

**Fig 1:**
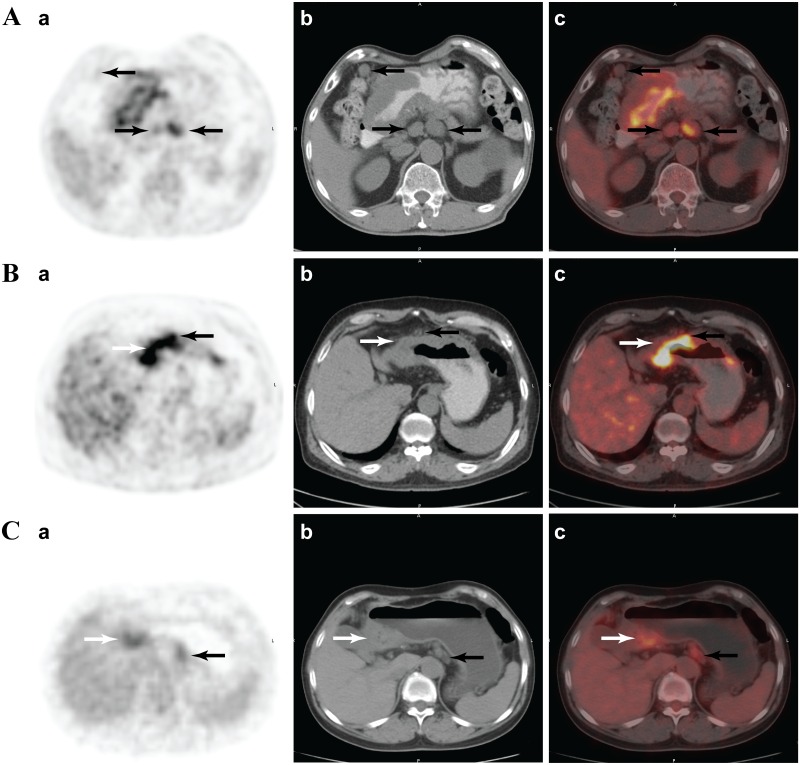
Analyses of regional lymph nodes. (A) A 57-year-old male with mucinous adenocarcinoma in gastric antral (white arrow) and metastatic lymph nodes (30/37). Three solitary lymph nodes with fluorodeoxyglucose (^18^F-FDG) uptake higher than surrounding fat tissue were evaluated as MPLN (black arrows). (B) A 62-year-old male with moderately to poorly differentiated adenocarcinoma in gastric antral and metastatic LN (3/17). A prominent nodular ^18^F-FDG uptake spot (black arrow) was observed in the stomach wall of the primary lesion (white arrow). The same level CT cross-section image showed a soft nodule adhesion in the gastric wall, and was counted as a MPLN. (C) A 62-year-old women with poorly differentiated adenocarcinoma in gastric antral and metastatic LN (34/52). An ^18^F-FDG uptake spot (black arrow) was noted in the rear gastric body and identified as a LN cluster. The cluster was counted as three MPLNs (a. Positron emission tomography imaging; b. the same slice with computed tomography imaging; c. positron-emission tomography and computed tomography fusion imaging).

For the regional lymph nodes, visually abnormal ^18^F-FDG uptake spots and lymph nodes around the stomach were carefully examined on multi-plane PET, and combined with CT and fusion images. Nodes with increased ^18^F-FDG uptake relative to the surrounding fat tissue were considered as MPLN (SUV_max_ >1.9). The highest nodal SUV_max_ was carefully measured and recorded. The numbers of solitary MPLN were counted and recorded with the combined CT image as a reference (Fig 1A). If lymph nodes were closely attached to the primary lesion on combined CT, and if corresponding PET showed that the primary tumour had nodular outward radioactive accumulation, they were counted as MPLN (Fig 1B). Cluster MPLN were counted with reference to the least number of sub-lymph nodes (usually defaulting to three) on the combined CT image (Fig 1C).

### Data analysis

Differences between variables were evaluated using the Fisher’s exact test and Wilcoxon rank test. We performed Spearman correlation analyses to determine the relationship between the numbers of metabolically positive lymph nodes and clinicopathological outcome, because these variables were not normally distributed (according to the one-sample Kolmogorov Smirnov normality test). The numbers of ^18^F-FDG-positive lymph nodes were constructed in receiver operating characteristic (ROC) curves to identify the optimal cut-off values for predicting N3. The diagnostic sensitivity, specificity, accuracy, positive predictive values, and negative predictive values were calculated according to standard definitions. Survival rates were estimated according to the Life Table and Kaplan-Meier using log-rank test. The Cox proportional hazards model was used to evaluate prognostic variables for multivariate survival analysis, and variable selection using stepwise regression. *P*-values below 0.05 were considered statistically significant. Statistical analyses were performed using the SAS 9.3 software package (SAS Institute Inc., Cary, NC, USA).

## Results

### Patient characteristics

Patient characteristics are presented in Table 1 (details shown in S1 Table). Thirty-eight were male and 12 were female, with a median age of 60 years (range 54-66). The localizations of gastric cancer in upper, middle and distal thirds were 9, 14, and 27, respectively. The predominant histological types (56%) were poorly differentiated adenocarcinoma and signet ring cell carcinoma/mucinous adenocarcinoma. A total of 78% (39/50 patients) underwent R0 resection, and 22% (11/50) were non-R0. TNM staging was 12% IB, 16% II, 50% III, and 12% IV. Histological lymph node positivity was 71.7% (33/46), and 34% (17/50) of patients were serum tumour marker-positive.

**Table 1:** Clinical and pathological features of patients with advanced gastric cancer (n = 50).

Parameter	Value
Gender	
Male	38 (76%)
Female	12 (24%)
Age (years)	60 (54, 66)
Pathologic type	
Well-differentiated	4 (8%)
Moderately differentiated	18(36%)
Poorly differentiated	19 (38%)
Signet ring cell/mucinous	9 (18%)
Tumour location	
Upper third	9 (18%)
Middle third	14 (28%)
Distal third	27 (54%)
Surgery	
Total gastrectomy	11 (22%)
Subtotal gastrectomy	13 (26%)
Distal gastrectomy	22 (44%)
Bypass	4 (8%)
Tumour invasion depth^a^	
T2	9 (18%)
T3	21 (42%)
T4	16 (32%)
Unknown	4 (8%)
Lymph node metastasis^a^	
N0	13 (26%)
N1	8 (16%)
N2	10 (20%)
N3	15 (30%)
Unknown	4 (8%)
Distant metastasis	6 (12%)
Peritoneal	3
Post-peritoneal lymph nodes	2
Organ	1
TNM stage^a^	
Stage IB	6 (12%)
Stage II	13 (26%)
Stage III	25 (50%)
Stage IV	6 (12%)
Surgical type	
R0	39 (78%)
Non-R0	11 (22%)
Tumour marker-positive	20 (40%)

^a^According to the 7th American Joint Committee on Cancer TNM classification.

By the end of follow-up, 29 (58%) patients had died, 18 were alive, and three were lost to follow-up. The median survival time was 32.57 months (range 3.0-94 months). The one-, three-, and five-year survival rates according to the survival curve were 84%, 48%, and 38%, respectively.

### FDG PET/CT findings

Visual inspection identified 72% (36/50) of the gastric cancers with positive FDG uptake and mean SUV_max_ was 9.32±4.99 (range 5.2–27.8), whereas 28% (14/50) showed low FDG uptake and mean SUV_max_ was 3.65±0.70 (2.7–4.5).

Among the 46 patients who had undergone lymphoectomy, histopathology confirmed 19 of 20 cases as truly positive for lymph metastasis by ^18^F-FDG-PET/CT. A false-positive was found in a patient with T3N0M0 disease, for whom pathological examination indicated node inflammatory hyperplasia. False-negative lymph metastasis was found in 14 patients. The false-negative rates for N1, N2 and N3 were 100%, 30%, and 20%, respectively. The sensitivity, specificity, positive predictive value (PPV), negative predictive value (NPV), and accuracy of PET/CT to detect lymph metastasis were 57.6% (19/33), 92.3% (12/13), 95.2% (20/21), 46.1% (12/26), and 69.6% (32/46), respectively.

### Evaluation of the numbers of MPLN and determination of the cut-off value

**Fig 2:**
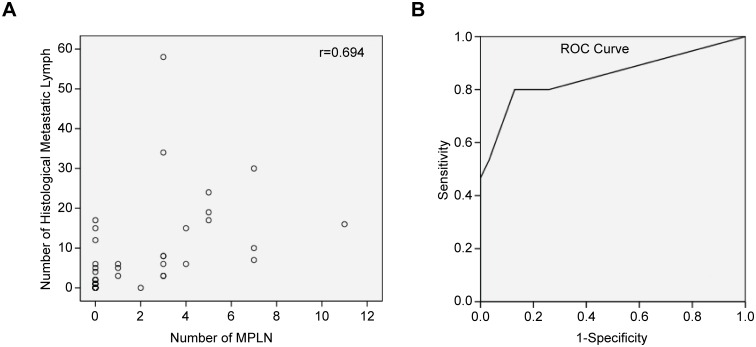
The relationship between the number of ^18^F-FDG-positive LN and histologically metastatic LN. (A) Scatter plot of the number of ^18^F-FDG-positive LN *vs*. the number of histologically metastatic LN (n = 46; Spearman’s correlation test, r = 0.694; p = 0.001). (B) ROC curves of the predictive value of the number of ^18^F-FDG-positive LN for N3; the area under the curve was 0.852 (95% CI: 0.712–0.991).

PET/CT imaging revealed a total of 103 MPLN in 23 (47.17%) patients, with a mean SUV_max_ of 4.87±2.67 (2.0–13.0), mean size of the largest nodes 1.62±1.02 cm (0.8–4.5 cm), and median number of 0 (0–3). Numbers of MPLN were moderately correlated with the numbers of histologically positive lymph nodes (r = 0.694, p = 0.001) (shown in Fig 2A) and weakly correlated with tumour invasion depth (r = 0.448, p = 0.002). No correlation was found between the numbers of MPLN and the primary focal SUV_max_ (r = 0.219, p = 0.126).

ROC curves were constructed to assess the predictive value of the numbers of MPLN for N3 staging. The area under the curve was 0.852(95% CI: 0.712–0.991) (shown in Fig 2B), and three MPLN was the best cut-off value. Using this cut-off value to predict N3, the sensitivity, specificity, PPV, NPV and accuracy were 80%, 87%, 75%, 90% and 85%, respectively (Table 2). Comparison of clinical pathologic features of the numbers of MPLNs ≤ 2 *vs*. ≥ 3 is presented in Table 3. MPLN ≥ 3 were found only in patients with stage III or IV cancer (73.7% stage III and 26.3% stage IV), and 96.8% (35/37) of patients with ≤ 2 MPNL underwent R0 resection. Only 47.4% (6/16) of patients with ≥ 3 ^18^F-FDG-positive lymph nodes underwent R0 surgery.

**Table 2:** Number of metabolically positive lymph node (MPLN) predicting N3 (n = 46).

Number of MPLN	Histological N3		Sum	Sensitivity	Specificity	PPV	NPV	Accuracy
	+	-						
+ (≥ 3)	12	4	16	80%	87%	75%	90%	85%
- (0–2)	3	27	30					
Total	15	31	46					

**Table 3:** Comparison of clinical pathologic features between difference number of MPLN group (≤ 2 and ≥ 3 groups).

Parameter	Number of MPLN ≤ 2 (n = 31)	Number of MPLN ≥ 3(n = 19)	P-value
Gender			
Male	9 (29.0%)	3 (15.8%)	0.3317
Female	22 (71.0%)	16 (84.2%)	
Age (years)	60.58 ± 11.42	59.68 ± 8.23	0.7674
Pathologic type			
Well-differentiated	4 (12.9%)	0 (0%)	0.8406
Moderately differentiated	9 (29.0%)	8 (42.1%)	
Poorly differentiated	12 (38.7%)	8 (42.1%)	
Signet ring cell/mucinous	6 (19.4%)	3 (15.8%)	
TNM stage			
Stage IB	6 (19.4%)	0 (0%)	< 0.0001
Stage II	13 (41.9%)	0 (0%)	
Stage III	11 (35.5%)	14 (73.7%)	
Stage IV	1 (3.2%)	5 (26.3%)	
Surgery type			
Non-R0	1 (3.2%)	10 (52.6%)	< 0.0001
R0	30 (96.8%)	9 (47.4%)	
Tumour marker level			
Positive	10 (32.3%)	7 (36.8%)	0.7398
Negative	21 (67.7%)	12 (63.2%)	
Mean SUV_max_ of primary	5.4 (3.9, 8.3)	7.6 (5.7, 11)	0.0455
Mean SUV_max_ of MPLN	3.6 (2.1, 3.7)	4.4 (3.5, 6.2)	0.1019

### Survival analysis

**Fig 3:**
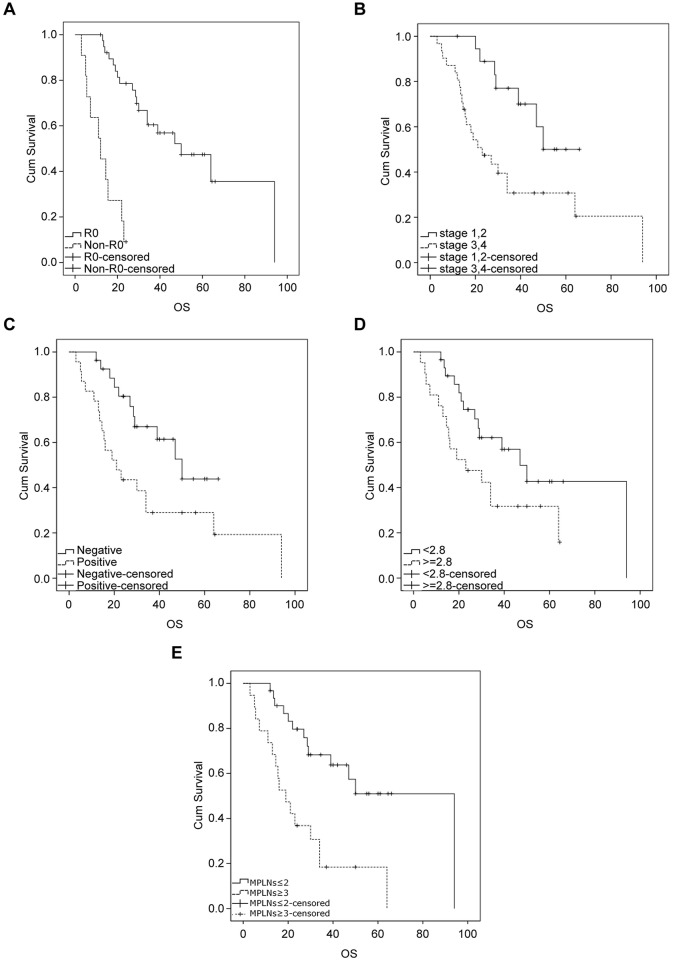
Kaplan-Meier survival curves for OS. (A) Surgery type (R0 *vs*. non-R0), *P* = 0.000; (B) TNM staging (I, II *vs*. III, IV), *P* = 0.016; (C) PET/CT LN (negative *vs*. positive), *P* = 0.029; (D) SUV_max_ of PET/CT LN (< 2.8 *vs*. ≥ 2.8), *p* = 0.021. (E) Number of ^18^F-FDG-positive LN (≤ 2 *vs*. ≥ 3), *P* = 0.0009. Tick marks indicate censored events.

Metabolic and clinicopathological variables for prediction of prognosis were tested in univariate analyses, and then significant variables were tested in multivariate analyses (presented in Table 4). The prognostic factors of surgery type (R0 *vs*. non-R0), pTNM stage (I, II *vs*. III, IV), PET/CT LN (positive vs. negative), SUV_max_ of PET/CT LN (< 2.8 *vs*. ≥ 2.8) (according to Song et al. [14]), and numbers of MPLN (≤ 2 *vs*. ≥ 3) were significantly associated with OS (Fig 3A–3E). The SUV_max_ of primary lesions (≤ 4.5 *vs*. > 4.5), tumour location (upper, middle *vs*. distal), serum tumour marker level (positive *vs*. negative), and histological grade (well, moderate *vs*. poor differentiation, SRC) were not significantly associated with OS.

**Table 4:** Univariate analyses and multivariate analysis for overall survival.

Factors	Median OS (months)	OS		
		HR	HR 95% CI	P value
Univariate analyses				
Ages (≤ 60 vs. > 60 years)	30/50	0.66	0.32–1.40	0.297
Sex (male vs. female)	34/39	1.02	0.41–2.53	0.969
Pathology (well, moderate vs. poorly, SRC)	47/34	1.04	0.49–2.22	0.925
Tumour location (upper, middle vs. distal)	29/34	1.20	0.56–2.54	0.641
Tumour marker (positive vs. negative)	30/39	1.42	0.67–3.05	0.362
Surgery type (R0 vs. non-R0)	50/12	9.40	3.65–24.21	< 0.0001
TNM staging (I, II, vs. III, IV)	66/23	2.90	1.22–6.88	0.016
SUV_max_ of Primary (≤ 4.5 vs. > 4.5)	47/29	1.80	0.76–4.28	0.185
SUV_max_ of PET/CT LN (< 2.8 vs.≥ 2.8)	50/19	2.51	1.15–5.23	0.021
PET/CT LN (negative vs. positive)	50/21	2.35	1.09–5.05	0.029
Number of MPLN (≤ 2 vs. ≥ 3)	94/19	3.60	1.69–7.69	0.0009
Multivariate analyses				
Surgery type (R0 vs. non-R0)		7.67	2.67–22.04	0.0002
Number of MPLN (≤ 2 vs. ≥ 3)		9.43	1.86–47.75	0.0067
SUV_max_ of PET/CT LN (< 2.8 vs. ≥ 2.8)		0.19	0.04–1.01	0.0514

*MPLN* metabolically positive lymph node

The Cox proportional hazards model was used to evaluate prognostic variables for multivariate survival analysis, and variable selection using stepwise regression. The numbers of MPLN (≥ 3 *vs*. ≤ 2), SUV_max_ of PET/CT LN (< 2.8 *vs*. ≥ 2.8), and surgery type (R0 *vs*. non-R0) were included in multivariate analyses. Surgery type (R0 *vs*. non-R0) (p = 0.002, HR 7.67, 95% CI: 2.67–22.04) and the numbers of MPLN (≥ 3 *vs*. ≤ 2) (p = 0.0067, HR 9.43, 95% CI: 1.86–47.75) were independent factors for poor OS.

## Discussion

The ability of metastasized tumour cells to invade lymphatic vessels is a more powerful prognostic factor than primary tumour features. Number of histological metastatic lymph nodes is closely related to patient survival. The UICC/AJCC (International Union Against Cancer and American Joint Committee on Cancer) lymph node staging system is based on the number of histological metastatic lymph nodes, and provides an effective means of gauging the prognosis of gastric cancer [15–17]. The five-year survival rate was 86.1% for N0 stage patients after surgery, but dropped abruptly to 58.1%, 23.3% and 5.9% for stages N1, N2 and N3, respectively [18]. However, histological information was lagging and retrospective, and did not contribute to the preferred treatment option. Pre-treatment knowledge of lymph node (LN) status would be helpful for determining prognosis and planning the optimal extent of lymphadenectomy or selecting patients who might benefit most from neoadjuvant chemotherapy. EUS, MDCT, MRI, and ^18^F-FDG-PET/CT are commonly used in clinical practice, but they do not achieve consistently high sensitivity and specificity for detecting LN metastasis [19]. Anatomy imaging has obvious limits when partly metastatic LNs are in normal size, and partly enlarged LNs were reactive hyperplasia. Although the sensitivity of ^18^F-FDG-PET/CT is lower than MDCT for lymph node involvement, there is a high specificity (95–100%) and positive predictive value (91–100%) [20–23], that is an advantage for predicting prognosis.

Coupe et al. [6] verified that in 97 patients at any stage of gastric cancer, ^18^F-FDG-PET lymph node positivity (*vs*. node negativity) was an independent and powerful predictor associated with inferior OS (HR 8.66, 95% CI 4.59–16.37, p<0.001). High nodal SUV_max_ (cut-off 2.8) measured by preoperative ^18^F-FDG-PET/CT was reported by Song et al. to be an independent prognostic factor for curative gastric cancer recurrence-free survival and OS. In this study, we also found that PET/CT lymph node positivity (*vs*. node negativity) and high SUV_max_ (≥ 2.8 *vs*. < 2.8) were significant in univariate analysis for predicting poor OS, but were not independent factors in multivariate analyses. We investigated an LAGC population undergoing surgical therapy, and those with early gastric cancer or who had evidence of distant metastatic gastric cancers were excluded from the study. Patients with no evidence of distant metastases until laparotomy was performed were included [S1 Table]. This sample selection differed from that of Coupe et al. and Song et al., and may have led to differing results.

Over-expression of Glut-1 is essential for cellular ^18^F-FDG uptake [24, 25]. Glut-1 over-expression in gastric cancer occurs when the tumour has already formed and gradually increases as the cancer progresses [25]. From this perspective, LN-positive ^18^F-FDG uptake should be associated with tumour progression and invasion of lymphatic vessels, and thus be indicative of prognosis. However, gastric cancer is of complex origin, with strong heterogeneity. The rates of Glut-1 over-expression were not associated with malignant grade of tumours, but were related to histological characteristics. Poorly differentiated adenocarcinoma and signet cell carcinoma often displayed low rates of Glut-1 over-expression [26]. Tumours containing mucus often show low FDG uptake [10], so SUV cut-off values limit prognostic judgment.

We explored the predictive value of the numbers of MPLN identified on ^18^F-FDG PET/CT. This is the first report of counting the number of lymph nodes in gastric cancer. This method was based on the high specificity and high positively predictive value of MPLN. Otherwise, PET/CT provides more accurate locations of ^18^F-FDG uptake through hybrid CT and fusion images. In this manner, PET/CT can minimise the likelihood of missing metastatic foci at low-level irradiative uptake and reduce misjudgements of both physiological uptake and LN location [27].

In this exploratory study, we found that number of MPLN is a useful prognostic marker in advanced gastric cancer pre-treatment evaluation. The numbers of MPLN were moderately correlated with the numbers of histological positive LN. This relationship was not linear, so there was no one-to-one relationship. The cumulate survival rate of patients with more MPLN (≥ 3) was significantly different from those patients with fewer MPLN (0–2); MPLNs (≥ 3) and surgery type (R0 *vs*. non-R0) were both independent factors of OS.

MPLN ≥ 3 was found only in patients with stage III or IV cancer; 52.6% of these patients underwent non-R0 resection, whereas only 3.2% of those with MPLN ≤ 2 did. Whether gastric cancer can achieve R0 resection is closely related to the surgeon's experience and ability, but distant metastasis is difficult to overcome. Detection of distant metastasis of gastric cancer by ^18^F-FDG-PET/CT is a valuable approach that can detect additional primary tumours or distant metastases that were not detected by conventional CT [28–32]. But early peritoneal metastasis and LN micro-metastases can still go undetected [28]. Approximately 20% of patients whose clinical and conventional radiological examinations indicated no distant metastases were found during surgery to have metastases [33, 34]. Hur et al. [12] reported that high SUV of the primary tumour (> 5) and positive ^18^F-FDG uptake in local LN during PET/CT could predict surgical failure to eradicate LAGC. Our findings indicate that large numbers of MPLN are correlated with advanced cancer stages and high risk of clinical occult distant metastasis.

It is known that gastric cancers containing mucous often display low FDG uptake, resulting in false negativity. Our method does not completely avoid the effects of false negativity, such as signet ring cell carcinoma, often presents false negative in primary lesion and lymph node.

Our study has certain limitations. Many factors influencing OS, in addition to the biological characteristics of tumours, include quality of initial surgical resection, patient's nutritional status, life-style, treatment after relapse, etc., and are associated with postoperative survival. In this study, we selected patients who were treated by the same surgical team to minimize variance of different surgeons. Due to the limited retrospective nature of the study and the small sample size to assess survival outcome, other factors were ignored. Therefore, statistical bias is inevitable. This investigation represents more of an exploratory analysis to guide future research. Further validation in a prospective study will be important, as well as addressing technical reproducibility and surgical/pathologic confirmation of the findings of PET/CT imaging.

We used a visual method to count the number of MPLN on PET/CT images. Although SUV_max_ of MPLN in the present study were greater than 1.9, we recommend that a visual approach of counting the positive LN is necessary to avoid confusion caused by noise, physiological uptake, and PET and CT morphology that were inaccurately matched. When only using a SUV threshold, such biases could result in false-positives or -negatives.

### Conclusions

In summary, MPLN is a useful marker for indicating inferior prognosis of LAGC. Quantifying the load of MPLN as the number of MPLN can provide additional prognostic information on LAGC. A gastric cancer with large numbers of MPLN indicated advanced stage disease, as well as possible of clinical occult distant metastasis. LAGC with MPLN ≥3 was a powerful predictive factor for poorer OS, and was one of the independent factors. Therefore, the number of MPLN may be a valuable reference for clinicians to design more rational treatment programs or multimodality therapy. The further clinical trials are needed.

## Supporting Information

S1 Table The patient’s clinical pathology and PET/CT performance details. (XLS)
